# Associations of high HDL cholesterol level with all-cause mortality in patients with heart failure complicating coronary heart disease

**DOI:** 10.1097/MD.0000000000003974

**Published:** 2016-07-18

**Authors:** Anping Cai, Xida Li, Qi Zhong, Minming Li, Rui Wang, Yingcong Liang, Wenzhong Chen, Tehui Huang, Xiaohong Li, Yingling Zhou, Liwen Li

**Affiliations:** a Department of Cardiology; b Guangdong Cardiovascular Institute, Guangdong Provincial Key Laboratory of Coronary Heart Disease Prevention, Guangdong General Hospital, Guangdong Academy of Medical Sciences, Guangzhou, China.

**Keywords:** cholesterol, heart failure, inflammation, prognosis

## Abstract

The aim of the present study was to evaluate the association between HDL cholesterol level and all-cause mortality in patients with ejection fraction reduced heart failure (EFrHF) complicating coronary heart disease (CHD).

A total of 323 patients were retrospectively recruited. Patients were divided into low and high HDL cholesterol groups. Between-group differences and associations between HDL cholesterol level and all-cause mortality were assessed.

Patients in the high HDL cholesterol group had higher HDL cholesterol level and other lipid components (*P* <0.05 for all comparison). Lower levels of alanine aminotransferase (ALT), high-sensitivity C-reactive protein (Hs-CRP), and higher albumin (ALB) level were observed in the high HDL cholesterol group (*P* <0.05 for all comparison). Although left ventricular ejection fraction (LVEF) were comparable (28.8 ± 4.5% vs 28.4 ± 4.6%, *P* = 0.358), mean mortality rate in the high HDL cholesterol group was significantly lower (43.5% vs 59.1%, *P* = 0.007). HDL cholesterol level was positively correlated with ALB level, while inversely correlated with ALT, Hs-CRP, and NYHA classification. Logistic regression analysis revealed that after extensively adjusted for confounding variates, HDL cholesterol level remained significantly associated with all-cause mortality although the magnitude of association was gradually attenuated with odds ratio of 0.007 (95% confidence interval 0.001–0.327, *P* = 0.012).

Higher HDL cholesterol level is associated with better survival in patients with EFrHF complicating CHD, and future studies are necessary to demonstrate whether increasing HDL cholesterol level will confer survival benefit in these populations of patients.

## 1 Introduction

Heart failure (HF) is a leading cause of morbidity and mortality around the world and coronary heart disease (CHD) is a major cause of HF.^[1,2]^ Despite percutaneous coronary artery intervention (PCI) and coronary artery bypass grafting (CABG) have substantially improved patients’ outcomes, nevertheless, the survival rate in patients with CHD complicated with ejection fraction reduced HF (EFrHF) is still extremely low with mortality rate up to 50% within 5 years since symptoms onset.^[3]^

High-density lipoprotein (HDL) cholesterol has well-known cardio-protective effects in CHD via mechanisms of enhancing cholesterol-reverse transport, anti-inflammatory, and antioxidative properties.^[4,5]^ In addition, it has been reported that among HF patients, as compared with survivors, HDL cholesterol level in nonsurvivors was significantly lower,^[6]^ suggesting that reduced HDL cholesterol level may portend poor survival rate in HF patients. Nevertheless, this study included patients with mixed etiologies of HF and whether reduced HDL cholesterol level is associated with all-cause mortality of EFrHF complicating CHD is unknown.

In the present study, we retrospectively recruited studied participants who had been angiographically diagnosed with CHD and echocardiographically diagnosed with left ventricular EF (LVEF) ≤35%. Results from the present study provide firsthand evidence regarding the impact of HDL cholesterol level on all-cause mortality in Chinese populations with EFrHF complicating CHD, and these results may also provide information for future prospective study in improving outcomes of patients with EFrHF complicating CHD via modifying HDL cholesterol level.

## 2 Methods

### 2.1 Studied participants

Studied participants were retrospectively selected and enrolled from Medical Document System of Guangdong General Hospital. The inclusion criteria comprised twofold as angiographically diagnosed with CHD treated with PCI and/or CABG and LVEF ≤35% as assessed by echocardiography. In brief, coronary artery significant stenosis is defined as ≥70% of stenosis of lumina of left anterior descending artery (LAD), left circumflex artery (LCA), or right coronary artery (RCA), and ≥50% of stenosis of lumina of left main (LM) artery. The present study was approved by the Clinical Research Ethic Committee of Guangdong General Hospital. Since this was a retrospective study, therefore no informed consent was obtained from studied participants. We searched studied participants in the Medical Document System of Guangdong General Hospital from the year of 2010 to 2015. At initial, 345 patients were selected according to above-mentioned inclusion criteria, after fully screening, those with HDL cholesterol data missing were excluded and a total of 323 patients were included into final analyses.

### 2.2 Data collection

Demographic, anthropometric, medical, and laboratory data were collected from Medical Document System of Guangdong General Hospital and were recorded in the case report form and were re-checked. Follow-up was conducted in the first quarter of 2016. In the processes of obtaining pre-specified clinical endpoints in terms of myocardial infarction, ischemic stroke, and cardiovascular and noncardiovascular mortality, we found that >90% of studied participants or their immediate relatives could not remember or elaborate above pre-specified clinical endpoints, neither the accurate date of events occurred. Therefore, we finally defined all-cause mortality as the present study's sole endpoint since vital status of each participant could be determined definitely. Moreover, most HF prognostic models have been developed for the outcome of all-cause mortality which is considered indisputable and unbiased.^[7]^

### 2.3 Statistical analysis

Continuous variables were expressed as mean ± SD when variables were normal distribution otherwise expressed as median and interquartile range. Normality of distribution for continuous variables was tested using the Kolmogorov–Smirnov test. Independent Student *t* test or nonparametric Mann–Whitney U test was used to assess between-group difference as appropriate. Categorical variables were expressed as number and frequency of cases, and between-group difference was analyzed by χ^2^-square analysis. Pearson correlation analysis was used to assess relationship between HDL cholesterol level with high-sensitivity C-reactive protein (Hs-CRP, using a Ln transformation as LnHs-CRP), alanine aminotransferase (ALT, using a Ln transformation as LnALT), and albumin (ALB), and Spearman rank analysis was used to assess relationship between HDL cholesterol level with cardiac function indexed by the New York Heart Association (NYHA) classification. Univariate regression analysis was used to assess the associations between factors and all-cause mortality, and forward stepwise strategy of multivariate regression analysis was used with an entry level of *P* <0.20 in univariate regression analysis. In brief, other factors known to have significant impact on HF survival such as age, diabetes, beta-blocker, angiotensin-converting enzyme inhibitor/angiotensin receptor blocker (ACEI/ARB), spironolactone, heart rate (HR), pulmonary artery pressure (PAP), and LVEF were also included to different models’ analyses as appropriate. Odds ratio (OR) and its associated 95% confidence interval (CI) was presented. All statistical analyses were conducted in SPSS 19.0 (SPSS Inc., Chicago, IL).

## 3 Results

### 3.1 General characteristics of studied participants

General characteristics of all studied participants are presented in Table 1. The mean age was 64.3 ± 10.8 years and males accounted for 84.2%, 48.0%, and 36.6% of studied participants had hypertension and diabetes, respectively, and notably nearly 60% had severe HF with NYHA III–IV classification. With respect to the number of coronary artery significant stenosis, 14.2%, 19.2%, 66.3%, and 21.7% had one-vessel, two-vessels, three-vessels, and LM plus three-vessels stenoses, respectively, and 92.6% had undergone PCI, 6.2% CABG, and 1.2% PCI plus CABG, respectively. With respect to laboratory examinations, mean serum level of total cholesterol (TC) was 4.54 ± 1.40 mmol/L, low-density lipoprotein (LDL) cholesterol 2.92 ± 1.19 mmol/L, HDL cholesterol 0.95 ± 0.28 mmol/L, ALB 33.8 ± 4.7 g/L, N-terminal pro B type natriuretic protein (NT-proBNP) 3792 (1744,7695) pg/mL, high-sensitivity troponin T (Hs-TNT) 20.4 (0.1,75.2) pg/mL, and Hs-CRP 9.9 (3.3,24.4) mg/L, respectively. And 97.2%, 93.5%, 81.7%, 69.3%, 64.4%, and 66.6% of studied participants were prescribed anti-platelet agents, statins, beta-blocker, ACEI/ARB, diuretic, and spironolactone at discharge, respectively. The mean LVEF was extremely low being 28.7 ± 4.6%, and mean mortality rate was extremely high being 53.6%.

**Table 1:**
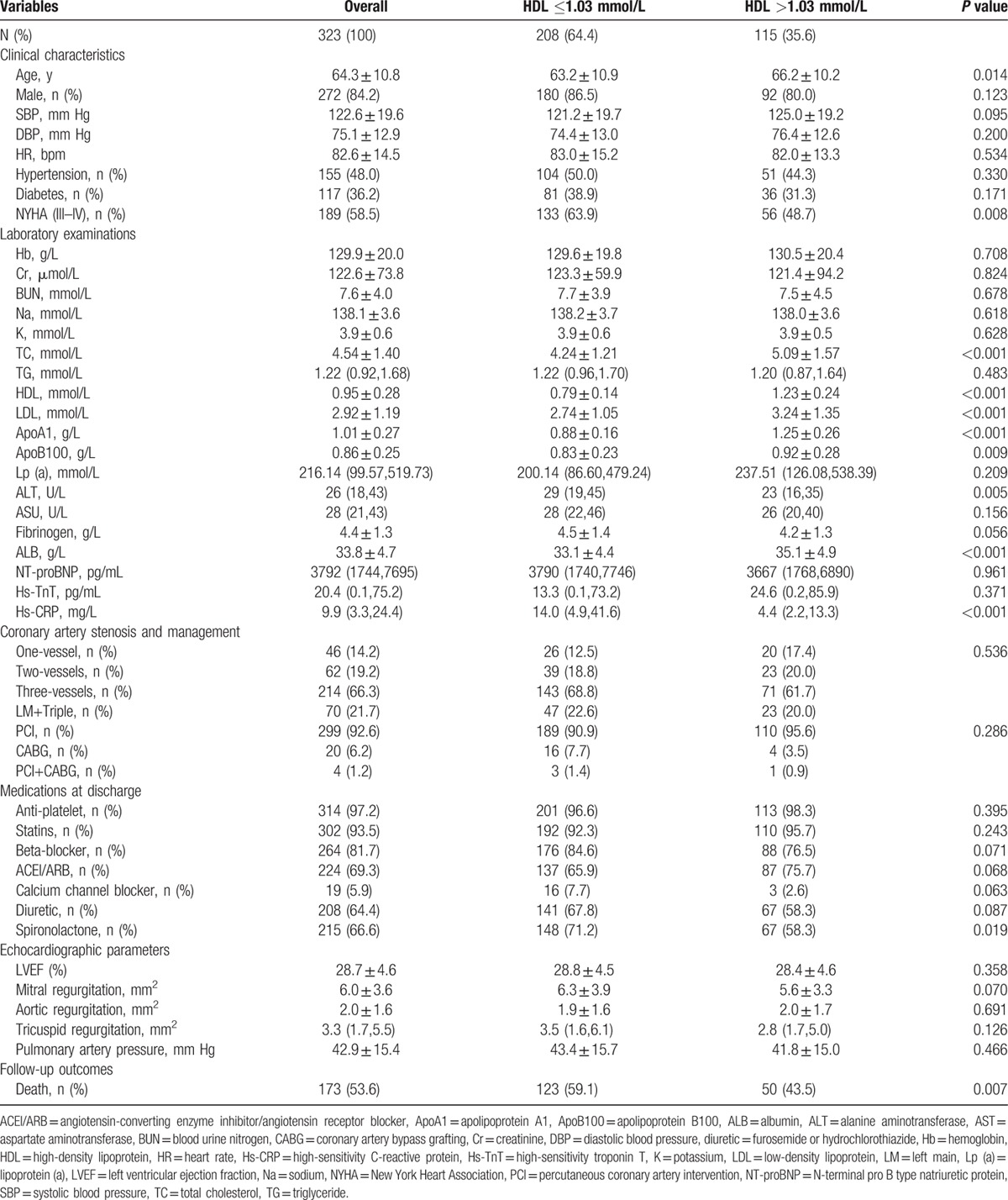
Comparisons by categories of HDL cholesterol levels.

### 3.2 Comparisons by categories of HDL cholesterol level

According to the criteria of the National Cholesterol Education Program (NCEP) Adult Treatment Panel (ATP) III,^[8]^ the cutoff value of low HDL cholesterol level is ≤1.03 mmol/L and therefore studied participants were divided into ≤1.03 mmol/L and >1.03 mmol/L groups. As shown in Table 1, there were substantial differences in clinical characteristics between these two groups. Compared with participants in the low HDL cholesterol group, participants in the high HDL cholesterol group were older and had less frequency of severe HF (*P* <0.05). Interestingly, apart from significantly higher HDL cholesterol level (1.23 ± 0.24 mmol/L vs 0.79 ± 0.14 mmol/L, *P* <0.001), other lipid components including TC, LDL cholesterol, apoA1, and ApoB100 were also significantly higher in the high HDL cholesterol group (*P* <0.05 for all comparisons). Between-group differences in ALT, ALB, and Hs-CRP were also observed (*P* <0.05 for all comparisons). No significant differences in the number of coronary artery significant stenosis, interventional, or surgical management of CHD and medications at discharge were observed except less frequency of spironolactone prescription in the high HDL cholesterol group (58.3% vs 71.2%, *P* = 0.019). Echocardiographic examination revealed that LVEF in both groups were comparable (28.8 ± 4.5% vs 28.4 ± 4.6%, *P* = 0.358), and both groups also had comparable degrees of mitral regurgitation (MR), aortic regurgitation (AR), tricuspid regurgitation (TR), and pulmonary arterial pressure (PAP) elevation. Notably, mean mortality rate in the high HDL cholesterol group was significantly lower (43.5% vs 59.1%, *P* = 0.007).

### 3.3 Lipid profiles comparison between survivors and nonsurvivors

Differences in individual lipid components were compared between survivors and nonsurvivors and only significant differences in HDL cholesterol level (1.00 ± 0.30 mmol/L vs 0.89 ± 0.25 mmol/L, *P* = 0.001) and ApoA1 level (1.05 ± 0.29 mmol/L vs 0.97 ± 0.23 mmol/L, *P* = 0.018) were observed.

### 3.4 Relationship between HDL cholesterol level and parameters of interest

**Figure 1:**
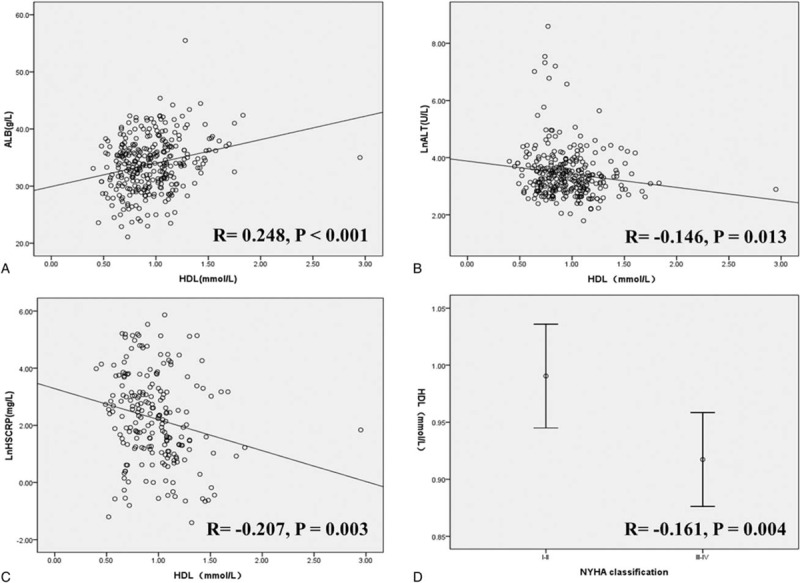
Relationship between HDL cholesterol level and parameters of interest. (Panel A) Relationship between HDL cholesterol level and ALB. (Panel B) Relationship between HDL cholesterol level and LnALT. (Panel C) Relationship between HDL cholesterol level and LnHs-CRP. (Panel D) Relationship between HDL cholesterol level and NYHA classification. ALB = albumin, HDL = high-density lipoprotein, LnALT = alanine aminotransferase, LnHs-CRP = high-sensitivity C-reactive protein, NYHA = New York Heart Association.

Relationship between HDL cholesterol level and parameters of interest were analyzed and as presented in Fig. 1, HDL cholesterol level was positively correlated with ALB level (*r* = 0.248, *P* <0.001), whereas inversely correlated with Ln-ALT and Ln-Hs-CRP, with a correlation coefficient of –0.146 (*P* = 0.013) and –0.207 (*P* = 0.003), respectively. Spearman rank correlation showed that HDL cholesterol level was inversely correlated with NYHA classification with a correlation coefficient of –0.161 (*P* = 0.004).

### 3.5 Logistic regression models

#### 3.5.1 Univariate regression analysis

As presented in Table 2, univariate regression analysis showed that there were a host of factors (including hypertension, higher NYHA classification, statins usage, higher serum levels of creatinine, ALT, NT-proBNP and Hs-CRP, lower serum levels of hemoglobin, HDL cholesterol, and ALB as well as more severe MR) were significantly associated with all-cause mortality in patients with HFrEF complicating CHD (*P* <0.20).

**Table 2:**
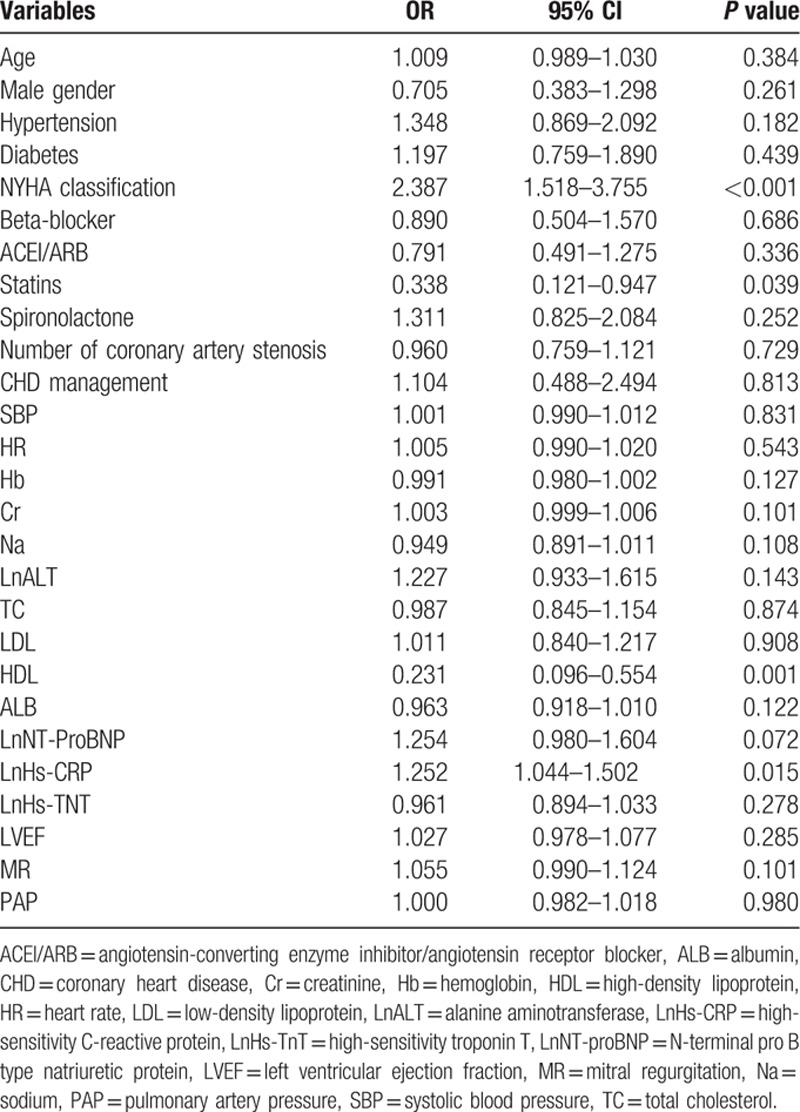
Univariate regression analysis of factors associated with all-cause mortality.

#### 3.5.2 Multivariate regression analysis

As presented in Table 3, multivariate regression analysis of HDL cholesterol effects on all-cause mortality revealed that after extensively adjusted for potential confounding variates, HDL cholesterol level remained significantly associated with all-cause mortality from Model 1 through Model 6 although the magnitude of association was weaken gradually.

**Table 3:**
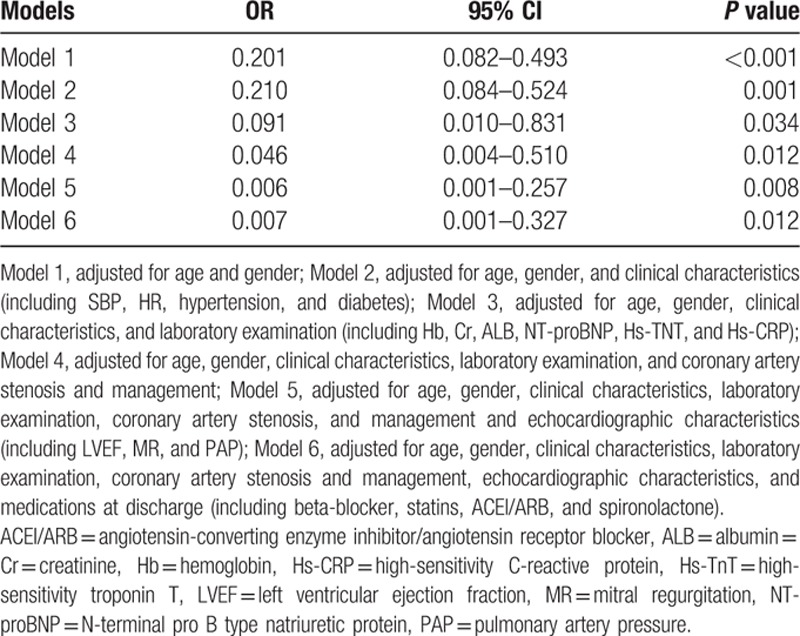
Multivariate regression analysis of HDL cholesterol effects on all-cause mortality.

## 4 Discussion

There are a substantial number of factors associated with prognosis of HF, and among them lipid profiles are the most attractive and intensively investigative. Previous clinical studies were largely focused on the associations between TC level and mortality,^[7,9,10]^ and the associations between HDL cholesterol level and mortality were less well studied. Our present study firstly provides information about the association between HDL cholesterol level and all-cause mortality of patients with EFrHF complicating CHD. Results indicate that higher HDL cholesterol level is associated with better survival outcome. In addition, HDL cholesterol level is positively correlated with ALB level, an important nutritional index, whereas inversely correlated with Hs-CRP, ALT, and NYHA classification. It is reported that HF is a chronic inflammatory status and most HF patients have elevated Hs-CRP level,^[11]^ suggesting that HDL cholesterol conferred survival benefits in HF patients might via its anti-inflammatory property, and inverse correlation between HDL cholesterol level and cardiac function indicated that patients with low HDL cholesterol level might be prone to developing more severe HF.

Dyslipidemia is a major risk factor of atherothrombotic disease such as CHD and reducing TC and LDL cholesterol levels by statins therapy has been demonstrated to be independently associated with favorable outcomes in CHD secondary prevention.^[12]^ Nonetheless, the efficacies of statins therapy on improving outcomes in HF patients are controversial and two large randomized controlled trials have showed that rosuvastatin therapy did not confer survival benefit in HF patients despite TC and LDL cholesterol levels were reduced by rosuvastatin therapy.^[13,14]^

Biologically and physiologically, cholesterol is an essential component of cellular membrane and varied hormones. Furthermore, cholesterol is a major energy resource particularly in condition like cachectic HF. In recent decades, many clinical studies have revealed that low TC level was independently associated with poor prognosis in HF, and underlying mechanisms are multifactorial. Indeed, most HF patients require more energy generation because of tachycardia and dyspnea which lead them to a cachectic condition. Furthermore, HF patients commonly suffer intestinal edema which adversely affects nutrition such as glucose and protein absorption. These pathophysiological alterations in HF not only lead patients to a metabolically demanding condition but also to a malnutrition status such as reduced ALB level in our studied patients (33.8 ± 4.7 mg/L). Moreover, circulating cholesterol-rich lipoprotein insufficiency results in endotoxin elevation leading to excessive inflammation^[15,16]^ such as increased Hs-CRP level in our studied patients (median level: 9.9 mg/L). Therefore, it is conceivable that higher cholesterol level is beneficial for HF patients.

Fewer studies have investigated the associations between HDL cholesterol level and prognosis of HF, and results of previous studies are inconsistent. For example, Sakatani et al^[6]^ reported that in HF patients with mixed etiologies, compared with nonsurvivors, HDL cholesterol level in survivors was significantly higher and no differences were observed in other lipid components. Nevertheless, Rauchhaus et al^[7]^ reported that compared with nonsurvivors, TC level in survivors was significantly higher and no significant difference in HDL cholesterol level between survivors and nonsurvivors was observed. Horwich et al^[10]^ also observed that in HF patients with 48% of ischemic etiology, both TC and LDL cholesterol levels but not HDL cholesterol level were significantly higher in survivors as compared with nonsurvivors. Different from these studies, we enrolled HF patients with solely ischemic etiology and our results indicated that compared with nonsurvivors, survivors appeared to have higher HDL cholesterol and ApoA1 levels, and no differences in other lipid components like TC and LDL cholesterol levels between survivors and nonsurvivors were observed. Conceivably, differences in study design, clinical characteristics, and HF etiology of studied patients might account for these discrepancies.

Compared with the low HDL cholesterol group, mortality rate in the high HDL cholesterol group was significantly lower, and after extensively adjusted for potential confounding factors including clinical characteristics, laboratory parameters, number and management of coronary artery stenosis, echocardiographic indexes, and medications at discharge, higher HDL cholesterol level remained independently associated with lower odds of all-cause mortality in patients with EFrHF complicating CHD. The mechanisms associated with survival benefits of higher HDL cholesterol level are multifactorial. On the one hand, patients with higher HDL cholesterol level might be at a better nutritional status as reflected by higher TC, LDL cholesterol, ApoA1, ApoB100, and ALB levels.^[17]^ On the contrary, HDL cholesterol has anti-inflammatory property and higher HDL cholesterol level is beneficial for ameliorating systemic inflammation as reflected by lower Hs-CRP level.^[18]^ Furthermore, HDL cholesterol has other cardio-protective properties like cholesterol-reverse transport and theoretically these efficacies may be also associated with survival benefits.

Liver is a major organ synthesizing cholesterol and therefore we evaluated the relationship between HDL cholesterol level and ALT level, a sensitive and specific marker of liver function. We observed that HDL cholesterol level was inversely correlated with ALT level. With respect to close relation between liver function and cholesterol synthesis, it was conceivable that impaired liver function might also play a role in prognosis of HF via mechanism of reducing cholesterols generation.

There are strengths and limitations of our present study. Different from previous studies, we solely enrolled HF patients with ischemic etiology which may avoid inconsistence of patients’ clinical characteristics. Moreover, this was the first study to evaluate the effects of HDL cholesterol level on all-cause mortality of HF in Chinese patients. However, since this was a retrospective study, therefore we could not infer a causal relationship between HDL cholesterol level and all-cause mortality. Second, we did not obtain data on long-term medications usage to adjust for the potential effect of medications treatment on outcome. Third, we did not adjust for body mass index or body weight for association between HDL cholesterol and study outcome because of lacking data on these two parameters. Both body mass index and body weight are closely correlated with lipid profiles including HDL cholesterol, therefore it was possible that the association between HDL cholesterol and study outcome might be over- or under-estimation which deserves further evaluation in prospective study. Finally, we had to alter our study design because of lacking data on pre-specified clinical endpoints and accurate date of event occurred.

## 5 Conclusion

Studies have showed a close relationship between total cholesterol level and prognosis of HF, however, the association between HDL cholesterol level and all-cause mortality of HF complicated by CHD is less well studied. Our present study reveals that higher HDL cholesterol level is associated with better survival outcome in these populations of patients. Understanding the association between HDL cholesterol level and prognosis of HF could provide new therapeutic target for improving clinical outcomes in HF patients.

## Acknowledgments

Authors thank Fang Fang Zeng, PhD, for performing statistical analysis of this article.
